# The Influence of the COVID-19 Pandemic on Current Teaching Methods, Training, and Perception Among Romanian Surgery-Oriented Students: Cross-Sectional Study

**DOI:** 10.2196/92294

**Published:** 2026-04-14

**Authors:** Ionut Dudau, Dumitru Sutoi, Bogdan Chiu, Raluca Radbea, George Marin, Anda Nicoleta Ciontos, Vlad Mulcutan-Chis, Daian Ionel Popa, Maria Sutoi, Andrei Catalin Zavragiu, Ovidiu Alexandru Mederle, Bogdan Nicolae Deleanu

**Affiliations:** 1 Doctoral School, Faculty of General Medicine Victor Babeș University of Medicine and Pharmacy Timișoara Timișoara, Timiș County Romania; 2 Department of Surgery, Emergency Discipline Victor Babeș University of Medicine and Pharmacy Timișoara Timișoara, Timiș County Romania; 3 Emergency Municipal Clinical Hospital, Timișoara Timișoara, Timiș County Romania; 4 Victor Babeș University of Medicine and Pharmacy Timișoara Timișoara, Timiș County Romania; 5 Institute of Cardiovascular Disease Timișoara Timișoara, Timiș County Romania; 6 Spitalul Clinic Judeţean de Urgenţă "Pius Brînzeu" Timişoara Timișoara, Timiș County Romania

**Keywords:** medical education, COVID-19 pandemic, medical students, medical training, post COVID-19 pandemic

## Abstract

**Background:**

The COVID-19 pandemic prompted rapid changes in medical education, accelerating the adoption of online and distance learning methods as alternatives to traditional teaching. While these approaches offered logistical advantages, students worldwide reported significant limitations, particularly in terms of motivation, clinical exposure, and hands-on skill acquisition. Despite the increased use of digital teaching during the pandemic, core educational objectives and the mission of medical training remained unchanged, emphasizing the continued importance of practical experience.

**Objective:**

This study aims to investigate the impact of the COVID-19 pandemic on current teaching methods in medical education and to explore students’ perceptions of online learning, telemedicine, artificial intelligence, and other modern educational alternatives.

**Methods:**

This observational, cross-sectional multicentric study surveyed a cohort of Romanian medical students using a self-developed 48-item online questionnaire distributed via social media. Data were collected over 6 weeks (February-March), yielding 451 responses, of which eligible participants included students in clinical years or preclinical students interested in surgical or orthopedic careers, with a heavy representation of the Medicine and Pharmacy University of Timisoara. Statistical analysis was performed using Microsoft Excel and JASP (University of Amsterdam; version 0.95.4).

**Results:**

A total of 436 responses were analyzed, with students favoring online or hybrid formats for lectures but preferring on-site teaching for practical training. Reduced patient interaction and limited skill acquisition were the main drawbacks of online practical education. Acceptance of hybrid learning correlated with more positive perceptions of teaching methods and a lower perceived desire to cheat.

**Conclusions:**

The COVID-19 pandemic brought significant changes to the way medicine is being taught in Romania, but it also brought a clearer picture for students and medical staff on how they want medical education to be done. Online cheating remains a significant challenge, but it is being tackled at the moment with different algorithms being tested.

## Introduction

The COVID-19 pandemic represented a period of transition and adaptation of teaching techniques, particularly within medical schools. Furthermore, it acted as a catalyst for the implementation of innovative teaching methods, particularly through the widespread adoption of online learning, which served as an alternative to the traditional teaching approaches used before this period [1-3]. Beyond curriculum reorganization [2], this period also facilitated innovation in medical education by accelerating the implementation of distance learning approaches, including webinars, newly adopted digital platforms, and virtual reality–based instructional methods [4,5].

Despite the advantages associated with online teaching methods, such as increased time flexibility, improved time efficiency, enhanced comfort due to the absence of commuting, and reduced financial costs, students reported several perceived disadvantages, including decreased motivation for learning, loss of direct clinical contact with patients and health care staff, and a reduction in hands-on practical experience [6,7]. Both the advantages and disadvantages were consistently highlighted by medical students across different regions of the world and countries with varying levels of socioeconomic development, including the United Kingdom, Iran, Pakistan, Saudi Arabia, as well as countries in Asia and Europe [6,8-10]. Moreover, similar findings were reported in Romania, where the overall student perception of the impact of the COVID-19 pandemic was predominantly negative (78%) [11].

When comparing the prepandemic and pandemic periods retrospectively, it can be stated that the fundamental teaching methods used in medical education have largely remained unchanged, owing to the essential need for practical experience acquisition and the development of a solid theoretical knowledge base closely integrated with clinical practice [12]. Furthermore, the mission of medical education remains constant regardless of context and represents the cornerstone for the development of future generations of physicians [13]. At the same time, retrospective analysis indicates that the use of distance learning methods increased significantly during the COVID-19 pandemic compared with the prepandemic period. Stoehr et al [14] emphasized that the purpose of online education is to achieve educational objectives rather than to replace them.

Tabatabai [15] emphasized the critical importance of students’ physical presence during training periods, highlighting the necessity of acquiring hands-on practical experience, as well as the limitations of virtual teaching methods in fostering practical competencies and clinical reasoning skills. Adaptability represents an indispensable quality for any future physician, as it requires creativity and the ability to respond to ever-evolving clinical contexts. In the postpandemic era, medical students must also acquire competencies related to telemedicine, as this modality has already become an integral component of health care delivery and is expected to expand substantially in the future [16].

In Romania, during the COVID-19 pandemic, the continuity of the educational process was ensured through distance learning modalities. Before this period, the use of telemedicine was limited. Following the pandemic, the adoption of online teaching methods increased significantly compared with the prepandemic era; however, their use has been reduced now that the pandemic period has passed.

The primary objective of this study is to analyze the effects of the COVID-19 pandemic on currently used teaching methods, particularly the use of online instructional approaches, and their impact on the training of future generations of surgical physicians, among a cohort of Romanian medical students. Additionally, this study aims to examine students’ perceptions regarding the use of artificial intelligence (AI), telehealth, and other modern alternatives for acquiring theoretical knowledge and practical skills. In addition to existing literature, this study specifically targets surgery-oriented students, a subgroup with distinct educational needs related to hands-on training and clinical exposure. Furthermore, it evaluates postpandemic perceptions regarding emerging educational tools such as AI and telemedicine within a Romanian context, where digital integration in medical education was limited before the pandemic.

## Methods

### Overview

In this observational, cross-sectional, multicentric study, a Google Form composed of 48 total questions, including demographic information, nominal data, ordinal data represented by Likert-type scales, and binary variables, was sent to medical students in Romania. At the beginning of February, responses were collected from students from all universities in Romania. The students have been selected using nonprobabilistic convenience sampling, with the ones who received the link and wanted to take part in the study being selected as the cohort to be analyzed. Students from the following universities have been contacted: “Victor Babes” University of Medicine and Pharmacy Timisoara, “Iuliu Hatieganu” University of Medicine and Pharmacy Cluj, “Dunarea de Jos” University of Galati, “Carol Davila” University of Medicine and Pharmacy Bucharest, “Ovidius” University of Constanta, “Lucian Blaga” University of Sibiu, University of Oradea, University of Medicine and Pharmacy of Craiova, Transilvania University of Brasov, and “Vasile Goldis” University of Arad. The last 2 universities only had 2 students, so to maintain a systematic standard across the multicentric study, the minimum threshold of 3 responders per university was established. In doing so, 4 responses did not meet this inclusion criterion. The questionnaire was sent using WhatsApp (Meta) and Instagram (Meta) groups, directly related to medical students in the universities previously mentioned. Response collection was made for a month and a half, until mid-March.

**Figure 1:**
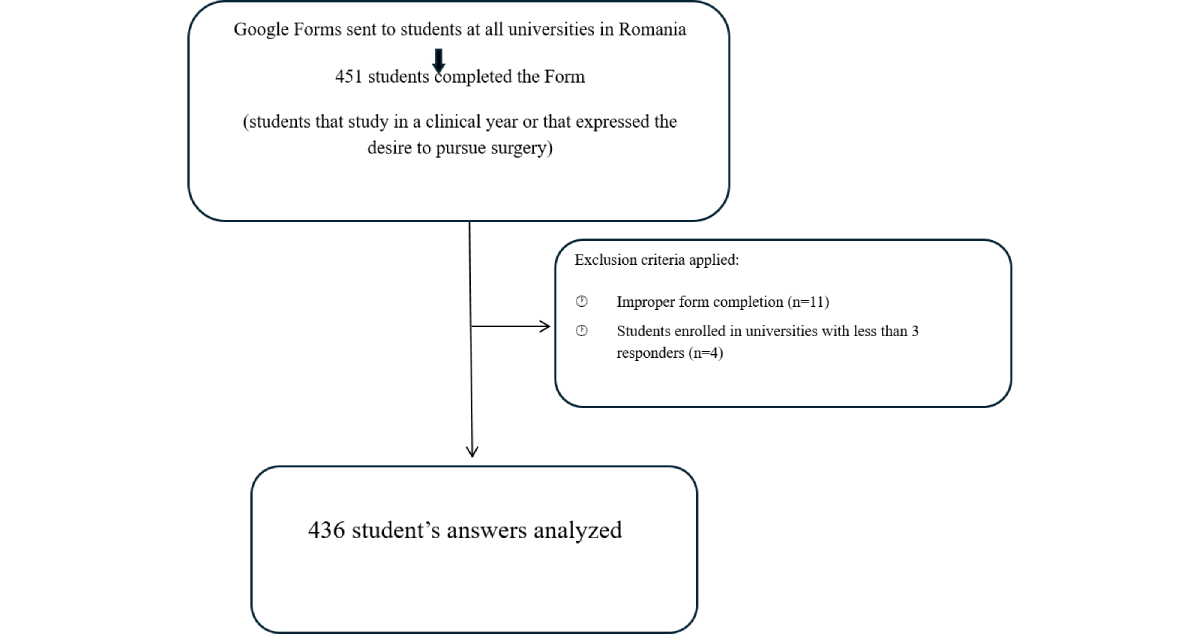
Flowchart of participants' selection.

After closing the form, a total of 451 students from Romanian medical universities responded to the form. The criteria for completing the form were to be enrolled in one Romanian medical university, to attend 1 of the 4 clinical years (third to sixth years) and to be interested in following a surgical career. If the students were enrolled in one of the first two years, they could complete the form only if they expressed their desire to follow a career in a surgical field. The objective of the study was clearly indicated in the form description, so the completion of the survey was considered an implicit confirmation of their interest in surgery. After eliminating the responses of the students that did not fit the inclusion criteria (either they did not complete the form properly or they were enrolled in universities where only 3 or fewer students responded to the form), the statistical analyses were done. The study flowchart is presented in Figure 1.

### Data Collection and Form Design

A self-developed questionnaire has been developed, with no other similar form being available in Romania. The questionnaire was developed under the supervision of experts (the surgical professor and the medical education expert in the author list), after a comprehensive literature review of similar studies to identify the key domains relevant to medical education that need further exploration of the subject. It included data collected for the demographic assessment of the study (age, gender, and university), Likert-type questions (ranging from 1 to 5, where 1 is the lowest level possible and 5 the highest), and multiple-choice questions that assessed the opinion of the students regarding online and onsite teaching methods. Additionally, the questionnaire addressed students’ perceived inclination toward cheating, the use of AI, and preferred examination methods. Before distributing the Google Forms, a small sample of 20 medical students from the Victor Babes University of Medicine and Pharmacy were selected to complete the form and evaluate the clarity and the technical functionality of the questionnaire. A test-retest procedure was performed after 1 week to evaluate response stability. Informed consent was obtained from all participants, with the first section of the Google Forms consisting of a general data protection regulation that needed the approval of the person completing the survey to advance to the other sections.

### Statistical Analysis

Statistical analyses were performed using Microsoft Excel (2024) and JASP (University of Amsterdam; version 0.95.4). Categorical variables were expressed as frequencies and percentages, while continuous variables were described using median (IQR), depending on data distribution. Data normality was assessed using the Shapiro–Wilk test, kurtosis, and skewness, with *P* values <.05 being considered as the threshold for rejecting the null hypothesis, thereby categorizing the data as non-normally distributed. Since most variables were ordinal or non-normally distributed, nonparametric tests were applied (the Wilcoxon signed-rank test and Spearman rho). The Kruskal-Wallis test was used to assess the differences in perception based on study year. Correlations between ordinal variables were evaluated using Spearman ρ correlation coefficient, with values *P*<.05 being considered statistically relevant, and the strength of the correlation being evaluated based on the value of rho. The internal consistency of the questionnaire was assessed using Cronbach alpha. The alpha has been calculated demonstrating acceptable reliability with a coefficient of α=0.742.

### Ethical Considerations

This study was conducted in accordance with the ethical principles outlined in the Declaration of Helsinki and adhered to the guidelines of the Committee on Publication Ethics. Ethical approval for this study was obtained from the Ethics Committee of the “Victor Babes” University of Medicine and Pharmacy Timisoara (approval number: 05/2020/03.02.2026_rev). Participation in the study was voluntary and anonymous. Before completing the questionnaire, all participants were provided with detailed information regarding the study objectives, data usage, and confidentiality. Informed consent was obtained electronically, as participants were required to agree to a consent statement before accessing the survey. No personally identifiable data were collected. All responses were anonymized and handled in accordance with the General Data Protection Regulation. Data were stored securely and used exclusively for research purposes. Participants were informed that they could withdraw from the study at any time before submission of the questionnaire, without any consequences. The Victor Babes University of Medicine and Pharmacy of Timisoara will be covering the publication costs for this research paper. This study did not receive any external funding.

## Results

In the final analysis of the database, only 436 out of the 451 total responders’ answers have been taken into consideration due to the exclusion criteria. Most of the students were female (340/436, 78%), and the most represented study year was the fourth year (115/436, 26.4%), closely followed by the fifth year students (101/436, 23.2%). With Romania having a 6-year medical school system, all study years have been well represented. Most students who answered the survey were enrolled in the “Victor Babes” University of Medicine and Pharmacy Timisoara (313/436, 71.8%; Table 1).

Table 2 showcases the descriptive statistics that were made on the Likert-type questions. The statistical analysis was made using the Shapiro-Wilk test, all calculations signaling a *P*<.001. The most notable finding was that students were willing to accept online teaching primarily for lectures, but in a hybrid manner, with a median of 4 compared to a median of 3 in the case of online-exclusive lectures. A hybrid way of teaching means that students would have lectures both onsite and online, based on the week and the decision of the professor. On the other hand, the responders consider that practical laboratories must be taught in an on-site fashion, not even the hybrid option being welcomed by students (median of 2 and IQR of 1-4). At the same time, students do not feel confident in the current ability of professors and medical teachers to use technology to its highest standard (median of 2 and IQR of 1-4).

Besides all of these, the desire to cheat is high in both online and onsite examinations. Online examination formats were perceived as offering more opportunities for academic dishonesty, which makes online testing a poor choice and should make professors be more alert about cheating opportunities. As can be seen, the desire of students to adopt more varied teaching methods is high, with a median of 5 (4-5).

The Wilcoxon signed-rank test revealed multiple statistically significant differences when performing the paired comparison. Online teaching looks to be a much better alternative for lectures rather than for practical training (*P*<.001; *r*=0.817). Students perceived much higher levels of academic dishonesty in an online format compared to in-person examinations (*P*<.001; *r*=0.893). Hybrid training seems to be much more accepted in the case of lectures rather than practical training (*P*<.001; *r*=0.922). The professor’s technological adaptation was linked with online lectures’ efficiency (*P*<.001; *r*=0.626) but not statistically significantly correlated with practical training’s efficiency (*P*=.43; *r*=0.051; Table 3)*.*

**Figure 2:**
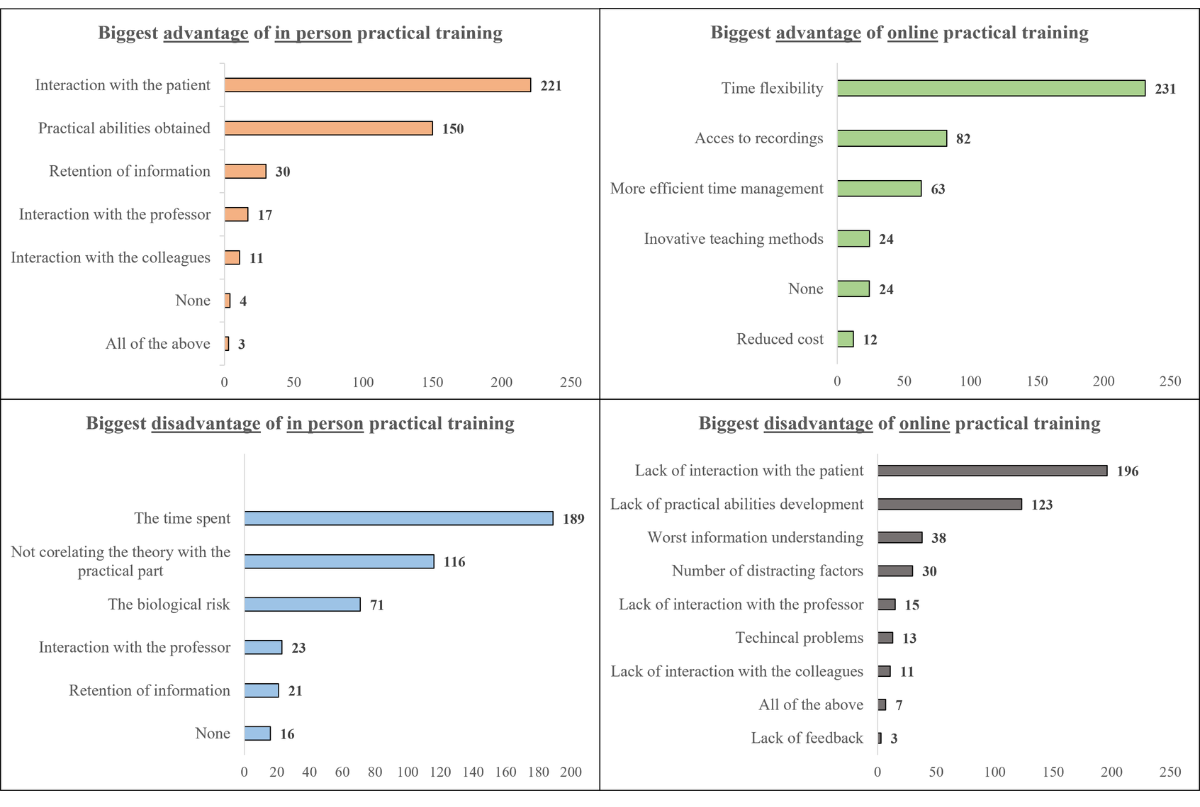
Distribution of perceived advantages and disadvantages of online vs in-person teaching of practical laboratories.

The practical side of medicine is essential to the development of future surgeons. It should be taught since medical school, with more practical methods being used. Figure 2 displays the advantages and disadvantages of online and in-person practical laboratories. For medical students in Romania, the time they spend at the university seems to be the most important factor in their choice of an online teaching method (231/436, 52.98%). On the other hand, we can see similar results between Figure 2 and Table 2, where the biggest disadvantage that students face when taking online practical laboratories is the lack of interaction with the patient (196/436, 44.95%) and the lack of practical abilities gained (123/436, 28.21%).

As can be seen in Table 4, most variables do not differ depending on study year. The Kruskal-Wallis test indicated that the study year has a minimal influence on student perception, with the effect size largely negligible. In this cohort, it seems that the students’ attitude toward online teaching, teaching methods, AI, and technology use does not differ from one study year to another. This indicates a consistency of perception between students.

**Figure 3:**
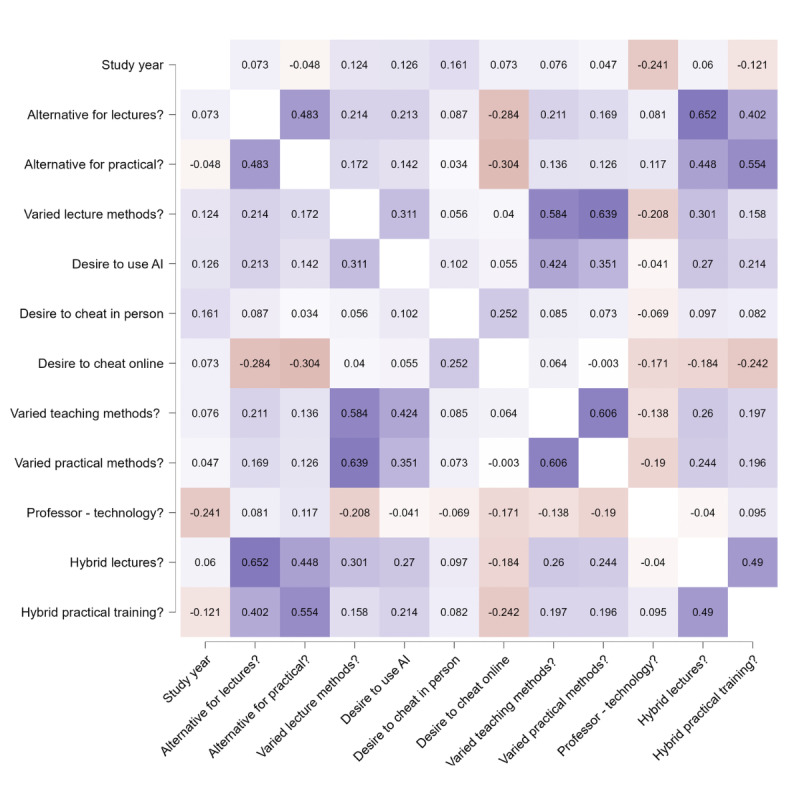
Heatmap of Likert-type data regarding perception of teaching methods, academic integrity, and technology use (Spearman ρ correlation). AI: artificial intelligence.

Figure 3 represents a heatmap of the correlations made between various parameters analyzed. The most powerful positive correlation is represented by the students who consider that online lectures and hybrid lectures represent a good alternative to traditional methods (*P*<.001, Spearman ρ=0.652). On the same note, students underline the importance of the teaching methods used, both in the hospital and in the lecture hall (*P*<.001, Spearman ρ=0.639). A strong correlation has also been observed between students who desire more varied teaching methods in general and those who desire more varied teaching methods for lectures (*P*<.001, Spearman ρ=0.584). The most pertinent negative correlation is between the students who consider online teaching a good alternative for practical laboratories and the desire to cheat online, even though this did not prove to be a statistically significant correlation. This might indicate, even though not statistically significant, that when students feel that they have more alternatives and are more involved in the educational process, their desire to cheat decreases (*P*<.001, Spearman ρ=–0.304).

**Table 1:** Demographic description of the population (n=436).

Variables		Values
**Sex, n (%)**		
	Male	94 (21.6)
	Female	340 (78)
	Other	1 (0.2)
	I prefer not to mention	1 (0.2)
**Year of study, n (%)**		
	1	55 (12.6)
	2	57 (13.1)
	3	56 (12.8)
	4	115 (26.4)
	5	101 (23.2)
	6	52 (11.9)
**University, n (%)**		
	UMFVBT^a^	313 (71.8)
	UMFIHCJ^b^	30 (6.9)
	UDJG^c^	28 (6.4)
	UMFCD^d^	25 (5.7)
	UOC^e^	24 (5.5)
	ULBS^f^	9 (2.1)
	UO^g^	4 (0.9)
	UMFCV^h^	3 (0.7)

^a^UMFVBT: “Victor Babes” University of Medicine and Pharmacy Timisoara.

^b^UMFIHCJ: “Iuliu Hatieganu” University of Medicine and Pharmacy Cluj.

^c^UDJG: “Dunarea de Jos” University of Galati.

^d^UMFCD: “Carol Davila” University of Medicine and Pharmacy Bucharest.

^e^UOC: “Ovidius” University of Constanta.

^f^ULBS: “Lucian Blaga” University of Sibiu.

^g^UO: University of Oradea.

^h^UMFCV: University of Medicine and Pharmacy of Craiova.

**Table 2:** Analysis of Likert-type questions regarding perception of online, hybrid, and onsite teaching methods.

Questions	Median (IQR)	Skewness	Kurtosis	*P* value^a^
Do you consider online teaching methods as an efficient alternative to lectures?	3 (1-5)	–0.21	–1.04	<.001
Do you consider online teaching methods as an efficient alternative for practical laboratories?	2 (1-4)	0.61	–0.77	<.001
Do you wish more varied methods were used in your lectures?	5 (4-5)	–1.40	1.40	<.001
Do you wish more varied methods were used in your practical laboratories?	5 (4-5)	–1.12	0.53	<.001
What level of desire do you consider exists when it comes to cheating in in-person exams?	3 (1-5)	0.17	–0.90	<.001
What level of desire do you consider exists when it comes to cheating in online exams?	5 (3-5)	–1.32	0.86	<.001
Would you agree to have hybrid lectures?	4 (2-5)	–0.618	–0.83	<.001
Would you agree to have hybrid practical laboratories?	2 (1-4)	0.518	–1	<.001
Do you wish to use more AI^b^ tools in your medical training?	4 (2-5)	–0.547	–0.426	<.001
Do you consider the professors to be ready to use technology to the highest standard?	2 (1-4)	0.613	–0.352	<.001
Do you wish more varied methods were used in your medical education?	5 (4-5)	–1.20	0.70	<.001

^a^*P* value determined using the Shapiro-Wilk test was used to assess data normality.

^b^AI: artificial intelligence.

**Table 3:** Paired comparison of student attitudes regarding teaching modalities.

Comparison	W^a^	*P* value	Effect size (*r*)^b^
Online lectures vs online practical training	31536	<.001^c^	0.817
Cheating in person vs cheating online	2674	<.001^c^	0.893
Hybrid lectures vs hybrid practical training	1264	<.001^c^	0.922
Professor’s technological adaptation vs online lectures	11306	<.001^c^	0.626
Professor’s technological adaptations vs online practical training	21700	.43	0.051
Desire to use AI^d^ vs cheating in person	12822	<.001^c^	0.432
Desire to use AI vs cheating online	44204	<.001^c^	0.609

^a^W: Wilcoxon signed rank statistic.

^b^*r*: rank-biserial correlation.

^c^Statistically significant values (*P*<.05).

^d^AI: artificial intelligence.

**Table 4:** Kruskal-Wallis test comparing students’ perception based on study year.

Questions	*H* ^a^	*P* value	Effect size (ε²)^b^
Do you consider online teaching methods as an efficient alternative to lectures?	5.23	.39	0.001
Do you consider online teaching methods as an efficient alternative for practical laboratories?	5.68	.34	0.002
Do you wish more varied methods were used in your lectures?	7.15	.21	0.005
Do you wish more varied methods were used in your practical laboratories?	4.61	.47	0.000
What level of desire do you consider exists when it comes to cheating in in-person exams?	12.17	.03^c^	0.017
What level of desire do you consider exists when it comes to cheating in online exams?	4.29	.51	0.000
Would you agree to have hybrid lectures?	6.64	.25	0.004
Would you agree to have hybrid practical laboratories?	6.64	.25	0.004
Do you wish to use more AI^d^ tools in your medical training?	10.48	.06	0.013
Do you consider the professors to be ready to use technology to the highest standard?	4.66	.46	0.000
Do you wish more varied methods were used in your medical education?	30.08	<.001^c^	0.058

^a^*H*: Kruskal-Wallis test statistic (factor: study year).

^b^ε²: epsilon-squared size effect.

^c^Statistically significant (*P*<.05).

^d^AI: artificial intelligence.

## Discussion

### Principal Findings

Unlike many studies conducted during the acute phase of the COVID-19 pandemic, this study evaluates postpandemic perceptions, offering insight into more stable student attitudes toward hybrid and digital education. Additionally, the focus on surgery-oriented students provides a more specific understanding of the limitations of online teaching in skill-dependent disciplines. The understanding of anatomy is essential for any surgeon. The use of AI is becoming more and more useful in the study of this subject, and many anatomists feel that in the future, there is a need to develop AI-related apps for the study of anatomy [17]. In our study, 65.6% (286/436) of the students declared that they use AI apps regularly. The most used AI apps are chatbots like ChatGPT (OpenAI) and Gemini (Google), with 80.3% (350/436) of students using such resources. Similar to the study cited, students showed a great desire to use more AI apps, with a median of 4 out of 5 (IQR 2-6).

AI is increasingly embedded in contemporary surgical practice. Current applications include image-guided navigation, intraoperative decision-support systems, robotic-assisted optimization, predictive modeling of postoperative outcomes, and automated image analysis [18]. In addition, machine learning algorithms are being developed to objectively assess surgical performance and provide structured feedback on technical skill acquisition [19]. Parallel to these clinical applications, AI is progressively influencing medical education. Large language models and AI-driven platforms support adaptive learning, rapid information synthesis, case-based self-assessment, and personalized feedback [20]. Evidence suggests that AI-assisted learning may enhance knowledge retention and clinical reasoning within competency-based frameworks [21,22]. Nevertheless, concerns persist regarding accuracy, overreliance, academic integrity, and potential impacts on critical thinking [23,24].

The substantial proportion of students in our cohort reporting regular AI use aligns with this global shift toward digitally augmented learning. Rather than replacing traditional instruction, AI appears to function as a complementary tool within blended educational models [25]. Consequently, the key challenge for medical schools is not whether to adopt AI, but how to integrate it responsibly while preserving academic rigor and ethical standards.

Bock et al [26] came to a very similar conclusion to ours when it comes to lectures and practical laboratories and the preferred method of teaching. In their study, 43.16% or respondents affirmed that they would prefer live lectures, where 49.5% of this cohort considered the online alternative an efficient one. On the other hand, a much higher percentage of students preferred online and prerecorded lectures in the study conducted by Watson et al [27]. Compared to the almost 50% ratios presented before, in this study, 74.04% (323/436) of students who were involved in this study considered pre-recorded lectures as extremely effective, showing much higher rates than in other studies.

One consistent finding of this study is the preference for in-person practical laboratories rather than online, as very few consider the hybrid approach to be effective. Teaching certain practical procedures can be done online, but only when practical kits are delivered to students or other physical materials are used [28,29]. In these 2 studies, practical suturing or dermatological kits were sent to students, but the teaching method was online, similar to during the COVID-19 pandemic, rather than fully online. Our study shows that this cohort of students prefers on-site practical laboratories. Only 22.5% (98/436) of the students thought online practical labs were effective for teaching an entire semester’s curriculum, with the main drawback being the lack of patient interaction (196/436, 45%). It’s evident that for the students involved in this study, acquiring practical skills must be done in person, with excellent results achieved through practical workshops in addition to the skills taught in medical school [30,31].

One major challenge that the online system encounters is the prevention of cheating in online exams. As Bilen et al [32] underline, cheating has to be expected in an online method of examination. That statement is backed by our study as well, with a median of 5 out of 5 on the Likert scale when asked about the desire to cheat online, compared to a median of 3 when considering in-person cheating. It was mentioned in Bilen et al’s [32] work that there are methods that can be implemented to mitigate the desire to cheat online, with inspiration from online chess communities. Besides diminishing the desire to cheat, it is very important to positively influence students and offer them the proper means to be educated. As we discovered, when students feel that they are more involved in the teaching process and have better educational alternatives, their desire to cheat decreases (*P*<.001, Spearman ρ=–0.304). This association suggests that perceived teaching quality may be related to attitudes toward academic dishonesty; however, causal relationships cannot be inferred from this study design. Therefore, the pedagogical quality appears to be negatively correlated with academic dishonesty. Different methods are being tested to detect online cheating better, and maybe one day eliminate it, as seen in the study performed by Alsabhan [33], which achieved an accuracy of 92%.

The COVID-19 pandemic brought the need for new, more modern teaching methods. These changes have been welcomed by the students. The median for the desire for more varied teaching methods used was 5 (IQR 4-6). These modern methods can be implemented both in person and online. For example, simulation-based learning has been used as an online tool, which students perceived as a better method compared to problem-based learning for online use, with a high degree of commitment to the teaching process being showcased by the students [34]. Problem-based learning seems to be one of the modern teaching methods that can’t be used in an online setting, as students consider it less engaging and deterring to the acquisition of clinical skills [35].

In Romania, as in many other countries, the COVID-19 pandemic led to an abrupt transition to fully online teaching across medical universities, reflecting the broader global shift toward emergency remote education [1,11]. Clinical rotations were suspended or significantly reduced, bedside teaching was replaced by virtual case discussions, and practical laboratory exposure was limited [8,36]. Assessment methods also shifted toward online examinations, frequently without standardized proctoring systems, raising concerns regarding academic integrity [32]. These structural changes appear to have had a lasting influence on students’ perceptions of teaching efficiency. The strong preference observed in our study for in-person practical laboratories may reflect the perceived loss of hands-on clinical exposure during pandemic restrictions [37]. Similarly, while theoretical content can be delivered digitally with acceptable satisfaction levels, experiential and patient-centered learning remains difficult to replicate virtually [38].

Like any teaching method, both in-person and online teaching methods have their advantages and disadvantages. In our study, 33.9% (148/436) of students say that the biggest advantage of online teaching is time flexibility, but in an Indian research paper, the ability to divide into smaller work groups was considered the biggest advantage [39]. These differences come from the various teaching methods used, the structure of medical school in that country, and the perception of technology. Saverino et al [40] had very similar findings to ours, where the main advantages mentioned were the better usage of time that has been saved by not travelling to university and back home. Besides, the biggest disadvantage mentioned by students in the cited paper is the lack of interaction between them, where only 2.5% of the students we asked considered it the main disadvantage. The study conducted by the teaching methods used and the availability of curriculum resources seem to be the most important aspects that influence students’ enthusiasm [41]. Moreover, students prefer a more interactive format of teaching rather than the old teaching methods used in medical school [42].

Future research should focus on longitudinal evaluation of hybrid teaching models combining structured in-person clinical exposure with evidence-based digital supplementation [43]. Objective performance metrics, rather than perception-based outcomes alone, should be incorporated to assess competency acquisition in postpandemic curricula. Additionally, further investigation into regulated AI integration within medical curricula is warranted, including institutional policies addressing ethical use, academic integrity, and faculty development [23,25]. Multicenter studies with proportional institutional representation would improve generalizability and support the development of coordinated national educational strategies.

### Study Limitations

This study presents a few limitations caused mainly by the subjective nature of the study. With students being asked from all medical universities in Romania, in any study year, differences in the method of teaching used will be seen, which brings bias about their past experiences. The vast majority of respondents were enrolled in one of the few universities mentioned, so the result may be influenced by this heavy lifting of one university. Despite the imbalance in university representation, this study offers one of the first multicentric perspectives on postpandemic medical education in Romania. The imbalance in university representation, combined with the use of social media for recruitment, limits the generalizability of the results. For future research, a more proportional representation of the universities in the Romanian educational system should be desired. Out of the total participants, 78% (340/436) are female, which can bring a degree of bias. Additionally, although the questionnaire demonstrated acceptable internal consistency and was pilot-tested, a full psychometric validation was not performed. Therefore, the findings should be interpreted within the context of an exploratory instrument. One other limitation is the distribution of the form, with students who are active on social platforms being favored.

### Conclusions

This study suggests a shift in pedagogical methodology preferences among the surveyed medical students post COVID-19 pandemic. It indicates that even though there are more options for students to study (the online options), they do not consider them as efficient. The only efficient alternative might be for lectures, where students would agree to hybrid lectures. Furthermore, students wish for more varied teaching methods, which would enhance their involvement in the teaching process. Finally, online examinations represent a major limitation of online teaching, which, at the moment, is not a viable option, with high levels of cheating being involved.

## Abbreviations

AI: artificial intelligence

## Data Availability

The dataset analyzed during this study is available from the corresponding author on reasonable request.

## References

[1] Rose S (2020). Medical student education in the time of COVID-19. JAMA.

[2] Ferrel MN, Ryan JJ (2020). The impact of COVID-19 on medical education. Cureus.

[3] Papapanou M, Routsi E, Tsamakis K, Fotis L, Marinos G, Lidoriki I, et al (2022). Medical education challenges and innovations during COVID-19 pandemic. Postgrad Med J.

[4] Savage DJ (2021). The COVID-19 pandemic as a catalyst for medical education innovation: a learner's perspective. FASEB Bioadv.

[5] Chick RC, Clifton GT, Peace KM, Propper BW, Hale DF, Alseidi AA, et al (2020). Using technology to maintain the education of residents during the COVID-19 pandemic. J Surg Educ.

[6] Dergham P, Saudagar FNI, Jones-Nazar CC, Hashim SA, Saleh K, Mohammedhussain AA, et al (2023). Medical students' perceptions towards online teaching during the covid-19 pandemic: a cross-sectional study from Saudi Arabia. Adv Med Educ Pract.

[7] Bączek M, Zagańczyk-Bączek M, Szpringer M, Jaroszyński A, Wożakowska-Kapłon B (2021). Students' perception of online learning during the COVID-19 pandemic: a survey study of polish medical students. Medicine (Baltimore).

[8] Dost S, Hossain A, Shehab M, Abdelwahed A, Al-Nusair L (2020). Perceptions of medical students towards online teaching during the COVID-19 pandemic: a national cross-sectional survey of 2721 UK medical students. BMJ Open.

[9] Maqbool S, Farhan M, Abu Safian H, Zulqarnain I, Asif H, Noor Z, et al (2022). Student's perception of E-learning during COVID-19 pandemic and its positive and negative learning outcomes among medical students: a country-wise study conducted in Pakistan and Iran. Ann Med Surg (Lond).

[10] Suzuki T, Murayama A, Kotera Y, Bhandari D, Senoo Y, Tani Y, et al (2022). Cross-country student perceptions about online medical education during the COVID-19 Pandemic. Int J Environ Res Public Health.

[11] Sutoi D, Bazavan CO, Sutoi M, Petrica A, Marza AM, Trebuian CI, et al (2023). The learning experience of Romanian medical students during the online teaching imposed by the COVID-19 pandemic. Adv Med Educ Pract.

[12] Lucey CR, Johnston SC (2020). The transformational effects of COVID-19 on medical education. JAMA.

[13] Lucey CR, Davis JA, Green MM (2022). We have no choice but to transform: the future of medical education after the COVID-19 pandemic. Acad Med.

[14] Stoehr F, Müller L, Brady A, Trilla A, Mähringer-Kunz A, Hahn F, et al (2021). How COVID-19 kick-started online learning in medical education-the DigiMed study. PLoS One.

[15] Tabatabai S (2020). COVID-19 impact and virtual medical education. J Adv Med Educ Prof.

[16] Cheng C, Humphreys H, Kane B (2022). Transition to telehealth: engaging medical students in telemedicine healthcare delivery. Ir J Med Sci.

[17] Abdellatif H, Al Mushaiqri M, Albalushi H, Al-Zaabi AA, Roychoudhury S, Das S (2022). Teaching, learning and assessing anatomy with artificial intelligence: the road to a better future. Int J Environ Res Public Health.

[18] Hashimoto DA, Rosman G, Rus D, Meireles OR (2018). Artificial intelligence in surgery: promises and perils. Ann Surg.

[19] Twinanda AP, Shehata S, Mutter D, Marescaux J, de Mathelin M, Padoy N (2017). EndoNet: a deep architecture for recognition tasks on laparoscopic videos. IEEE Trans Med Imaging.

[20] Kung TH, Cheatham M, Medenilla A, Sillos C, De Leon L, Elepaño C, et al (2023). Performance of ChatGPT on USMLE: potential for AI-assisted medical education using large language models. PLOS Digit Health.

[21] Masters K (2019). Artificial intelligence in medical education. Med Teach.

[22] Chan KS, Zary N (2019). Applications and challenges of implementing artificial intelligence in medical education: integrative review. JMIR Med Educ.

[23] Tlili A, Shehata B, Adarkwah MA, Bozkurt A, Hickey DT, Huang R, et al (2023). What if the devil is my guardian angel: ChatGPT as a case study of using chatbots in education. Smart Learn Environ.

[24] Cotton DRE, Cotton PA, Shipway JR (2023). Chatting and cheating: ensuring academic integrity in the era of ChatGPT. Innovations in Education and Teaching International.

[25] Wartman SA, Combs CD (2018). Medical education must move from the information age to the age of artificial intelligence. Acad Med.

[26] Bock A, Peters F, Winnand P, Kniha K, Heitzer M, Lemos M, et al (2021). One year of COVID-19 pandemic: a cross sectional study on teaching oral and maxillofacial surgery. Head Face Med.

[27] Watson C, Templet T, Leigh G, Broussard L, Gillis L (2023). Student and faculty perceptions of effectiveness of online teaching modalities. Nurse Educ Today.

[28] Sutoi D, Cindrea AC, Popa DI, Trebuian CI, Williams C, Sutoi M, et al (2025). Impact of hands-on workshops on future medical students' motivation, confidence, and career aspirations: an observational study. J Med Life.

[29] Sutoi D, Popa DI, Cindrea CA, Trebuian CI, Williams C, Sutoi M, et al (2025). The impact of a one-day multidisciplinary workshop on medical students' self-assessed confidence, knowledge, and teamwork skills: a pre-post study. Adv Med Educ Pract.

[30] Wittbecker LM, Pham C, Wohlgemuth LK, Hoang MA, Bandholz TC, Schuh S, et al (2022). [Digital and innovative teaching in dermatology : Practically oriented teaching online]. Dermatologie (Heidelb).

[31] Tandon D, Gupta A, Patel R, Shukla A, Gill SS, Patel RE, et al (2025). The impact of online teaching curricula on undergraduate basic surgical skills acquisition. Surg Open Sci.

[32] Bilen E, Matros A (2021). Online cheating amid COVID-19. J Econ Behav Organ.

[33] Alsabhan W (2023). Student cheating detection in higher education by implementing machine learning and LSTM techniques. Sensors (Basel).

[34] Mamakli S, Alimoğlu MK, Daloğlu M (2023). Scenario-based learning: preliminary evaluation of the method in terms of students' academic achievement, in-class engagement, and learner/teacher satisfaction. Adv Physiol Educ.

[35] Adongo PR, Epuitai J, Mpagi JL, Nekaka R, Lyagoba I, Odula J, et al (2023). "No PBL is better than online PBL": qualitative exploration regarding the perceived impact of online problem-based learning on nursing and medical students' learning during COVID-19 lockdown. Res Sq.

[36] Ahmed H, Allaf M, Elghazaly H (2020). COVID-19 and medical education. Lancet Infect Dis.

[37] Compton S, Sarraf-Yazdi S, Rustandy F, Radha Krishna LK (2020). Medical students' preference for returning to the clinical setting during the COVID-19 pandemic. Med Educ.

[38] Wilcha RJ (2020). Effectiveness of virtual medical teaching during the COVID-19 crisis: systematic review. JMIR Med Educ.

[39] Kumari A, Rani S, Bara MP (2022). A Study on the perception of medical students using online teaching during covid -19 pandemic. J Family Med Prim Care.

[40] Saverino D, Marcenaro E, Zarcone D (2022). Teaching histology and anatomy online during the COVID-19 pandemic. Clin Anat.

[41] Chen M, Ye L, Weng Y (2022). Blended teaching of medical ethics during COVID-19: practice and reflection. BMC Med Educ.

[42] Watchmaker J, Gonzales EC, Larson AR (2019). Interactive teaching and repeat exposure maximize medical student satisfaction but do not promote long-term retention of dermatologic knowledge. Dermatol Online J.

[43] Goh PS, Sandars J (2020). A vision of the use of technology in medical education after the COVID-19 pandemic. MedEdPublish (2016).

